# Drivers for Livestock-Associated Methicillin-Resistant Staphylococcus Aureus Spread Among Danish Pig Herds - A Simulation Study

**DOI:** 10.1038/s41598-018-34951-1

**Published:** 2018-11-16

**Authors:** Jana Schulz, Anette Boklund, Nils Toft, Tariq Halasa

**Affiliations:** National Veterinary Institute, Technical University of Denmark, Kgs. Lyngby, Denmark

## Abstract

To gain insight into the rapid increase in the number of livestock-associated Methicillin-resistant *Staphylococcus aureus* (LA-MRSA)-positive herds in Denmark, we developed an individual-based Monte Carlo simulation model. We aimed to assess whether transmission of LA-MRSA via pig movements could explain the observed increase in the number of positive herds in Denmark, and to evaluate the effect of other between-herd transmission mechanisms. Pig movements alone were not sufficient to mimic the observed increase in LA-MRSA-positive herds in Denmark in any of the modelled scenarios. The model identified three factors that played important roles in the between-herd spread of LA-MRSA: (1) the within-herd dynamics, (2) the frequency and effectiveness of indirect transmissions, and (3) unexplainable introduction of LA-MRSA to swine herds. These factors can act as starting points for the development of LA-MRSA control programs in pig herds in order to limit the risk of its transmission to humans.

## Introduction

*Staphylococcus aureus* (*S*. *aureus*) is a ubiquitous bacterium in humans and animals, and a common cause of minor skin infections that do not usually require treatment. However, both *S*. *aureus* and Methicillin-resistant *S*. *aureus* (MRSA), which is resistant to beta-lactam antimicrobials, can lead to severe infections that could result in death, especially in individuals with a suppressed immune status. Livestock-associated MRSA (LA-MRSA) was first described in the Netherlands in 2005, and a special clonal complex, CC398, was identified shortly after^1^. LA-MRSA has been found in humans, pigs and other animal species since 2005^2–9^. In 2006, the pathogen was found in isolates originating from Danish pig farms^10^. A survey conducted in 26 European countries by the European Food Safety Authority (EFSA) in 2008 found 3% of Danish production herds, but no Danish breeding herds, positive for LA-MRSA type CC398^2^. However, a national survey conducted in 2014 found a prevalence of 63% in breeding herds and 68% in slaughter pig herds^11^. LA-MRSA has received enormous media attention due to its zoonotic potential, both in Denmark and beyond the borders.

Danish pig production has a pyramidal structure^12,13^. LA-MRSA-positive breeding sites at the top of the pyramid pose a risk of spreading the pathogen via pig movements to production sites in the centre and down to sites like slaughterhouses at the bottom of the pyramid. Several studies have indicated that the transmission of LA-MRSA via animal movements is one of the main drivers for its spread among pig herds^14–16^. Other transmission routes described are: air, housing environment, rodents, companion animals, vehicles and humans^17–23^. Understanding the spread of LA-MRSA among pig herds is essential to developing meaningful control programs for the pig production sector. The main goal of action plans made by the Danish authorities is to reduce the number of LA-MRSA-positive pigs and herds, thereby reducing the risk of transmission to humans.

Simulation models are widely used as a way to inform decision making. They present an opportunity to test a hypothesis without experiments or in cases where experimental studies are not possible^24,25^. They cover a wide range of applications such as modelling the within-host infection dynamics, disease-spread modelling within or between populations, and evaluating control strategies to support decision makers in the control of infectious diseases^24^. Since EU regulations require identification and registration of animals and animal movements^26^, data are available on a large scale and can be used as input in disease-spread models. Furthermore, if a pathogen is mainly spread through animal movements, the movement data can be used retrospectively to investigate its spread.

An individual-based Monte Carlo simulation model of the spread of LA-MRSA within and between pig herds was developed, integrating available data on Danish pig herds and registered pig movements among these herds. The purpose was to examine what might have influenced the rapid spread of LA-MRSA among Danish pig herds during the period 2006 to 2015. The aims were to: (1) assess whether transmission of LA-MRSA via registered pig movements alone could account for the observed increase in prevalence in Denmark, and (2) evaluate the effect of other transmission mechanisms. The results should assist in creating an agenda for the control of LA-MRSA in Danish pig herds.

## Materials and Methods

### Data background for the simulation model (input data)

In Denmark, information on pig holdings and pig movements are registered in the Central Husbandry Register (CHR)^27^, which is owned by the Ministry of Environment and Food. A dataset containing information on 18,648 pig herds and 7,678,851 movements among pig herds in Denmark was extracted for the study period 1^st^ January 2006 to 31^st^ December 2015. The dataset is described in detail by Schulz *et al*.^13^, and data preparation for the simulation model is described in the Supplementary Information. Several herds could be owned by the same farmer and thus constitute one holding. The study was performed at herd level, but information on ownership was retained to aid the modelling of LA-MRSA spread among herds with the same owner.

Herds registered as: (1) breeding and multiplier herds (breeding sites), (2) production herds, organic or free-range pig herds (merged into one category) and weaner herds (production sites), and (3) hobby herds (hobby sites) were used in our analysis, as well as the respective movements among these herds. End-of-production sites (e.g. 133 herds registered as slaughterhouses and 2,961,979 movements to slaughterhouses) were excluded because it was assumed that there would be no spread of pathogens back into the production chain from these. The remaining herd types and respective movements (2,657 herds and 116,386 movement records) were excluded because it was assumed they would have little impact on LA-MRSA spread due to the low number of registered herds and pig movements (e.g. zoo, experimental facilities) or because of missing information on the herd structure and age group of moved pigs (e.g. traders, trade herds). Any movements for which the number of pigs was not recorded were excluded (5,595 movement records). This left 49 herds that never moved pigs, and these were also excluded. In total, 12,874 herds and 993,474 movements were included in our study. If a herd changed its holding type to one not included in this study, the herd was assumed to be closed for the considered year, but kept active in the model for all other years.

### LA-MRSA screening results in Danish pig herds

In 2008, the European Food Safety Authority (EFSA) conducted an LA-MRSA survey in 26 European countries^2^. In subsequent years, national Danish surveys reported a rapid increase in the prevalence of LA-MRSA in breeding herds and slaughter pig herds^11^ (Table 1).

**Table 1:** Overview of LA-MRSA screenings performed in Danish pig herds between 2008 and 2014.

Year [reference]	Description	Number of tested herds	Estimated LA-MRSA prevalence in % [95% confidence interval]
2008^2^	Five dust samples were collected from the immediate environment of breeding pigs in the herd then pooled and tested for LA-MRSA	95 breeding herds: 198 production herds:	0.0 [0.0–3.2] 3.5 [1.8–7.1]
2010^39^	Pools of five nasal swab samples were taken from five slaughter pigs in five different pens	99 herds with slaughter pigs:	16.2 [9.5–24.9]
2011^40^	Pools of five nasal swab samples were taken from five slaughter pigs in five different pens	79 herds with slaughter pigs:	16.5 [9.1–26.5]
2014^11^	Pools of five nasal swabs per herd	70 breeding herds: 205 production herds:	63.0 [50.5–74.1] 68.0 [60.9–74.1]

### Simulation model

**Figure 1:**
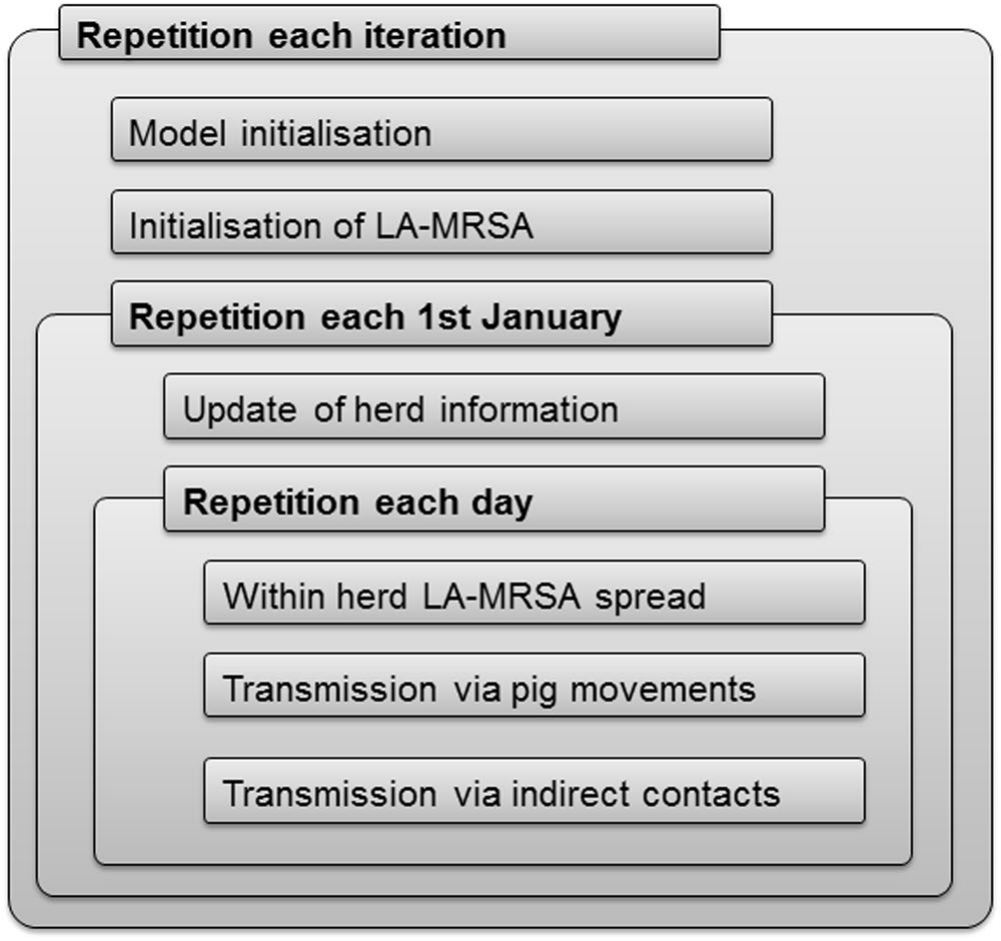
Overview of the structure of the livestock-associated Methicillin-resistant Staphylococcus aureus model.

An individual-based Monte Carlo simulation model for the spread of LA-MRSA among pig herds was developed (Fig. 1). Information on pig herds and movement data were read from input files at the beginning of each simulation run. After LA-MRSA was initialised, three processes within each time step were modelled to simulate within-herd dynamics, transmission via pig movements and indirect transmission among herds. For each simulation repetition and on each simulated day, information on positive herds was stored in files for later interpretation.

#### Model initialisation

In the first step, information on herds and movement data was sourced. Four additional files were read by the model, and these contained: (1) simulation parameters (Table 2); (2) minimum, most likely (mode) and maximum values defining probability distributions (PERT distributions) for transmission and cure rates (Supplementary Information, Table S5); (3) a distance matrix containing the distances between all herds; (4) distance probabilities of contact among herds for the different types of contacts (Supplementary Information, Table S6).

In the next step, a herd matrix was generated containing the following information for each herd: (1) an individual identification number (1–12,874); (2) the herd type registered in the CHR in 2006, set to zero for herds that were inactive (i.e. no movements registered) in 2006; (3) the simulated LA-MRSA status (equal to zero); (4) the number of sows, weaners and finishers registered (or estimated numbers as described in the Supplementary Information); (5) the number of LA-MRSA-positive sows, weaners and finishers (equal to zero); (6) status of antimicrobial usage based on the herd information (Supplementary Information); (7) transmission rates; (8) cure rates. Transmission and cure rates were randomly chosen from PERT distributions for each herd based on information from the literature^28^ (Supplementary Information, Table S5).

To initialise LA-MRSA in the simulation, it was necessary to define the number of herds and the herd types where LA-MRSA would be seeded. Additionally, the number of LA-MRSA-positive pigs and the age group of the first LA-MRSA cases were set. In the selected herds, ten LA-MRSA-positive sows were initialised. If fewer than ten sows were available, LA-MRSA was initiated in the weaner or finisher section. Several scenarios to mimic different situations in 2006 and different introductions at later points in time were tested (Supplementary Information, Table S7). In 2006, LA-MRSA was seeded exclusively in large herds (number of sows, weaners and finishers above *th*_*small*_), whereas when it was initialised in later years, small herds could also randomly be chosen.

The model simulated daily time steps, while the herd matrix was updated on a yearly basis, e.g. herd type, number of animals and antimicrobial usage were updated according to the input data. Transmission and cure rates were only updated if the herd type changed between two consecutive years. The number of positive animals in each age group was calculated for the next year based on the prevalence in the previous year. If the herd type changed between years, the LA-MRSA status and number of positive sows, weaners and finishers was set to zero, with the assumption that cleaning and disinfection were carried out before changing the herd type.

#### Modelling disease spread within a herd

**Figure 2:**
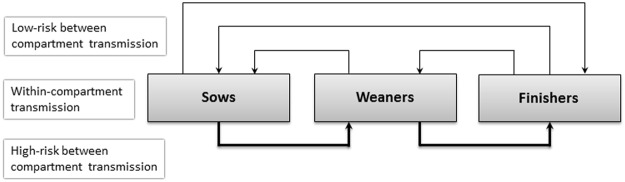
Schematic description of the implemented within-herd LA-MRSA spread.

The following processes were implemented and used in the order they are described:

1. *Environment-related recurrence*. For herds that had previously been LA-MRSA positive but had no LA-MRSA-positive pigs on the simulation day (due to self-cleaning or selling all positive pigs), the LA-MRSA status of the herd was set to negative in the model. Nevertheless, depending on the time since the status changed to negative, an exponentially decreasing probability of one pig reverting to LA-MRSA positive status due to environmental contamination was simulated (Supplementary Information).
  If the herd type changed between two consecutive years, this recurrence due to environmental contamination was still possible.
2. *Self-cleaning of individual pigs*. In positive herds, pigs could clear themselves randomly at any time according to the cure rates for sows, weaners and finishers within the herd (Supplementary Information, Table S5).
3. *Within-herd dynamics*. A three-compartment within-herd model was implemented with transmission within the three compartments and high- and low-risk transmission routes between the compartments for sows, weaners and finishers (Fig. 2). Homogenous mixing within each compartment was assumed. High-risk transmission was assumed between subsequent compartments within the production line to mimic LA-MRSA transmission due to pig movements within the herd. A lower risk of LA-MRSA transmission was assumed in the opposite direction to the production line, representing indirect transmission mechanisms e.g. via humans or equipment.

The transmission parameters published by Broens *et al*.^28^ (Supplementary Information, Table S5) take into account the pen structure in the compartments, which was not reflected in our model. The development of the within-herd prevalence in the first year after initialisation of LA-MRSA therefore showed a rapid increase (Supplementary Information, Figures S1–S4). In order to slow down the within-herd spread to reflect pen structure, a time-dependent shifting function was introduced (as described in Equation 5 in the Supplementary Information), representing the speed and steepness of LA-MRSA spread as modelled by Sørensen *et al*.^29^.

In herds with fewer than a certain number of animals (default value:th_small_ = 200), only one compartment was modelled and homogeneous mixing was assumed. No time-dependent shifting was used for the transmission rates in small herds. Supplementary Information Figure S5 shows the within-herd prevalence in production herds with fewer than 200 animals for (a) transmission rates adapted from Broens *et al*.^28^ and (b) time-shifted transmission rates for (1) herds using high-risk antibiotics, and (2) herds not using high-risk antibiotics.

All subsequent scenarios were run with time-shifted transmission rates adapted based on Broens *et al*.^28^ and Sørensen *et al*.^29^.

#### Modelling disease spread among herds

*Spread via animal movements:* Danish movement data do not include information on the age group of pigs moved between herds. As differences in prevalence can be expected among age groups, it was necessary to define movement types reflecting the age groups for each movement. The movement types were based on the number of registered sows, weaners and finishers in both the sending and receiving herd (Supplementary Information).

Transmission via pig movements was included based on the movement data and types. The number of LA-MRSA-positive pigs that were moved to the receiving herd was calculated using a binomial process representing the total number of pigs that were moved and the prevalence within the source compartment. The herd size in both sending and receiving herds was kept constant, with the assumption that whenever pigs were moved in, a similar number of pigs were moved out. However, the number of LA-MRSA-positive pigs was updated on a daily basis for both herds. If there were no weaners registered in the sending herd, but the age group of pigs moved out of this herd was estimated to be weaners, the prevalence in the sow section was used. Nevertheless, the number of positive sows in this section was not updated.

*Spread via indirect contacts:* Other transmission routes such as vehicles (feeding and abattoir trucks) and humans (veterinarians, technicians and visitors) were pooled as indirect contacts among herds.

Two pathways of indirect transmission were modelled: (1) distance-dependent transmission from LA-MRSA-positive herds via e.g. humans moving between farms, and (2) transmission related to abattoir movements. Indirect transmission related to human and abattoir movements was restricted to contacts within the same day of visits to several herds. For each LA-MRSA-positive herd *h*, the number of indirect contacts was calculated based on a Poisson distribution using *λ*_*in*_, *λ*_*out*_, or ${\lambda }_{a}^{h}$. It was determined whether each indirect contact was effective based on the PERT distributions *Prob*_*in*_, *Prob*_*out*_ or *Prob*_*a*_, given in the input file (Table 2). In the case of an effective indirect contact, a herd was randomly chosen based on the read-in distance probability tables (Supplementary Information, Table S6).

**Table 2:** Overview of simulation parameters and default values. PERT distributions were defined as transformation of the Beta distribution with minimum (min), maximum (max) and most likely value (mode) and a mean $\,\mu =\frac{min+4\cdot mode+max\,}{6}$.

Variable name	Default value	Description	Reference
**Modelling disease spread within a herd**			
*Environment-related recurrence*			
*envir*	1	Switch on/off environment-related recurrence within-herd (0/1)	
*α*_*envir*_	2.5	*α* in probability function for environment-related recurrence	Within-herd module validation
*Within-herd dynamics*			
*tds*	1	Switch on/off time-dependent scaling of within-herd transmission	
*x*	50	Midpoint s-shape transmission curve	Within-herd module validation
*k*	0.05	Steepness of s-shape transmission curve	Within-herd module validation
*hm*	1	Switch on/off homogeneous mixing in small herds (0/1)	
*th*_*small*_	200	Herds sizes below or equal to *th*_*small*_ are categorised as small herds – indicating random mixing within herd	Assumption
**Modelling disease spread among herds**			
***Spread via indirect contacts***			
*ic*	1	Switch on/off transmission via indirect contacts (0/1)	
*λ*_*in*_	0.256	Average daily probability of indirect contact originating from an LA-MRSA-positive indoor herd	Adjusted based on Boklund *et al*.^41^
*λ*_*out*_	0.1864	Average daily probability of indirect contact originating from an LA-MRSA-positive outdoor herd	Adjusted based on Boklund *et al*.^41^
*prob*_*in*_	PERT (min = 0.001, max = 0.01, mode = 0.005071)	Probability of infection via contact from an LA-MRSA-positive indoor herd	Expert opinion
*prob*_*out*_	PERT (min = 0.001, max = 0.01, mode = 0.0035)	Probability of infection via contact from an LA-MRSA-positive outdoor herd	Expert opinion
*prob*_*a*_	PERT (min = 0.001, max = 0.01, mode = 0.004714)	Probability of infection via abattoir movements	Expert opinion

In addition, transmission among herds with the same owner was modelled to represent shared workers or equipment (Supplementary Information).

#### Validation and convergence

The model was internally validated using three methods^30^: (1) face validity – using flow charts to ask people with an insight into the system whether the conceptual model was reasonable (Fig. 1, Figures S4–S8), and in the case of within-herd validation, whether the within-compartment prevalence represented a meaningful range, (2) the tracing method - following individual herds over time to determine whether the logic was correct, and (3) sensitivity analysis - evaluating the effect of changes in the fixed parameters. In addition, external validation was conducted by comparing the predicted prevalence of LA-MRSA by the model to the observed prevalence of LA-MRSA from the screening results in Danish swine herds (Table 1).

We assessed the number of simulation repetitions required before the variance in the total number of LA-MRSA-positive herds stabilised on 31^st^ December 2015 (Supplementary Information, Figure S9). Although convergence was reached after 250 iterations, the model was run with 500 iterations per scenario to cover any extra variability in the different initialisation scenarios and in the sensitivity analysis.

A sensitivity analysis was performed based on a scenario in which LA-MRSA was initialised in 100 active large production herds on 1^st^ January 2006, as well as one breeding and multiplier herd and 100 production herds on 1^st^ January 2009. The model was run with different settings (e.g. without environment-related recurrence, Table 3) and all fixed parameters were halved and doubled (Table 3). The effects of changes were evaluated by changes in the within-herd dynamics and the total number of infected herds 10 years after the first initialisation of LA-MRSA

**Table 3:** Overview of scenarios run in the framework of the sensitivity analysis. All default values (Table 2) were halved and doubled. For PERT distributions (*prob*_*in*_, *prob*_*out*_, *prob*_*a*_), minimum, mode and maximum values were halved and doubled.

Acronym	Description	Values
**Modelling disease spread within a herd**		
***Environment-related recurrence***		
NoEnvir	No environment-related recurrence within-herd	*envir* = 0
Envir	Variation of *α*_*envir*_	${\alpha }_{envir}\in \{1.25,5\}$
***Within-herd dynamics***		
NoTDS	No time-dependent shifting of within-herd transmission	*tds* = 0
Mid	Variation of *x*	$x\in \{25,75\}$
Steep	Variation of *k*	$k\in \{0.025,0.1\}$
NoHM	No homogeneous mixing in small herds	*hm* = 0
THhm	Variation of *th*_*small*_	$t{h}_{small}\in \{100,400\}$
**Modelling disease spread among herds**		
***Spread via indirect contacts***		
NoIC	No indirect contacts	*ic* = 0
IC_in/out_	Only distance-dependent indirect contact for indoor and outdoor herds	
IC_a_	Only indirect contact related to abattoir movements	
IC_sw_	Only indirect contact related to herds with the same owner	
FreqIC_in/out_	Variation of *λ*_*i*_ and *λ*_*o*_	${\lambda }_{i}\in \{0.128,0.512\}$ ${\lambda }_{o}\in \{0.0932,0.3728\}$
ProbIC_in/out_	Variation of *Prob*_*in*_ and *Prob*_*out*_	$Pro{b}_{in}\in \{PERT(0.0005,0.005,0.0025355),$ $PERT(0.002,0.02,0.010142)\}$ $Pro{b}_{out}\in \{PERT(0.0005,0.005,0.00175),$ $PERT(0.002,0.02,0.007)\}$
ProbIC_a_	Variation of *Prob*_*a*_	$Pro{b}_{a}\in \{PERT(0.0005,0.005,0.002357),$ $PERT(0.002,0.02,0.009428)\}$

Data processing, simulation modelling and graphical presentation of results were performed in R version 3.2.2 - “Fire Safety”^31^.

## Results

### Animal movements alone (initialisation scenarios)

**Figure 3:**
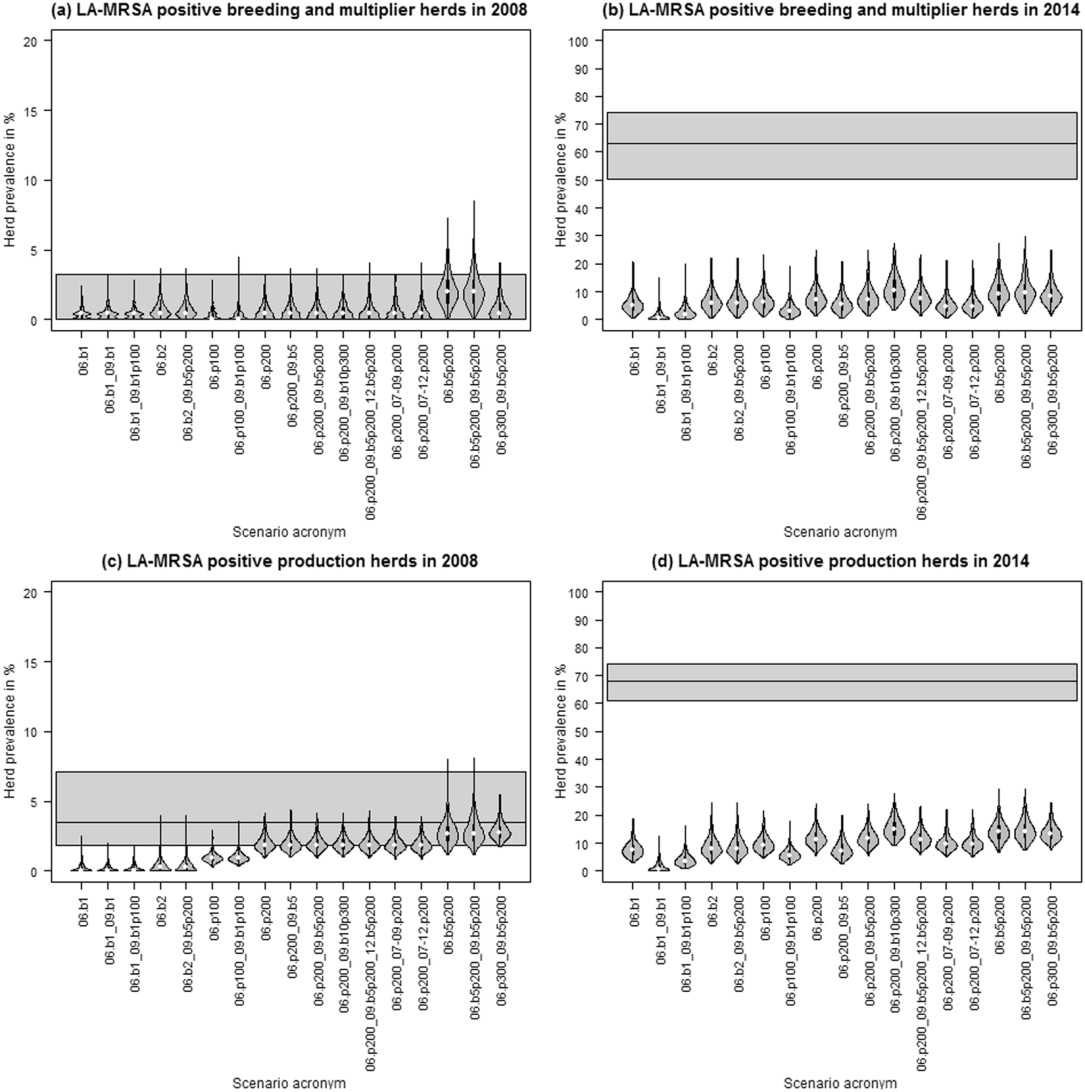
Herd prevalence in breeding and multiplier herds (**a**,**b**) and production herds (**c**,**d**) on 31^st^ December 2008 and 31^st^ December 2014 resulting from simulated LA-MRSA transmission via pig movements only, based on 500 iterations. Time-shifted transmission rates adapted from Broens *et al*.^28^ were used. The horizontal black line and grey area mark the observed prevalence and 95% confidence interval of the LA-MRSA screenings performed in Danish swine herds in 2008 and 2014. Example acronyms: 06.b5p200 – initialisation in five breeding and multiplier herds and 200 production herds in 2006, 06.b5_07-09.p200 – initialisation in five breeding and multiplier herds in 2006 and 200 production herds each year from 2007 to 2009.

To assess whether the transmission of LA-MRSA via pig movements alone could account for the observed pattern of increased prevalence in Denmark, the model was run with 17 initialisation scenarios (Supplementary Information, Table S7). Based on the assumption that LA-MRSA was already established in 2006, five scenarios were set up which introduced LA-MRSA in varying quantities of breeding and multiplier and/or production herds in 2006. Additionally, in 12 scenarios, LA-MRSA was introduced at later points in time to mimic new introductions. The simulated proportion of LA-MRSA-positive holdings in 2008 and 2014 was compared to the observed results of LA-MRSA screenings performed in Denmark (Table 1). In all scenarios, LA-MRSA was initialised on 1^st^ January 2006 in a number of breeding and multiplier herds and/or production herds (Supplementary Information, Table S7). Furthermore, scenarios were initiated to mimic further introduction of LA-MRSA in subsequent years. The predicted prevalence in breeding and multiplier herds matched the results of LA-MRSA screenings performed in Danish pig herds in 2008 (Table 1), but was lower than the observed prevalence in 2014 (Fig. 3). Animal movements could explain the observed prevalence in production herds in 2008 for certain scenarios, for example when the spread was initiated in many herds in 2006, or combined with initialisation in breeding herds. However, none of the scenarios could explain the observed prevalence in 2014 using animal movements alone (Fig. 3 and Supplementary Information, Figure S6.).

### Animal movements and indirect contacts (initialisation scenarios)

In the next step, all initialisation scenarios were run using pig movements and indirect contacts as transmission mechanisms and the between-herd prevalence on 31^st^ December 2015 was compared. In all scenarios, the modelled prevalence was higher when indirect contacts were added, compared to simulations in which transmission was modelled via animal movements alone (data not shown).

**Figure 4:**
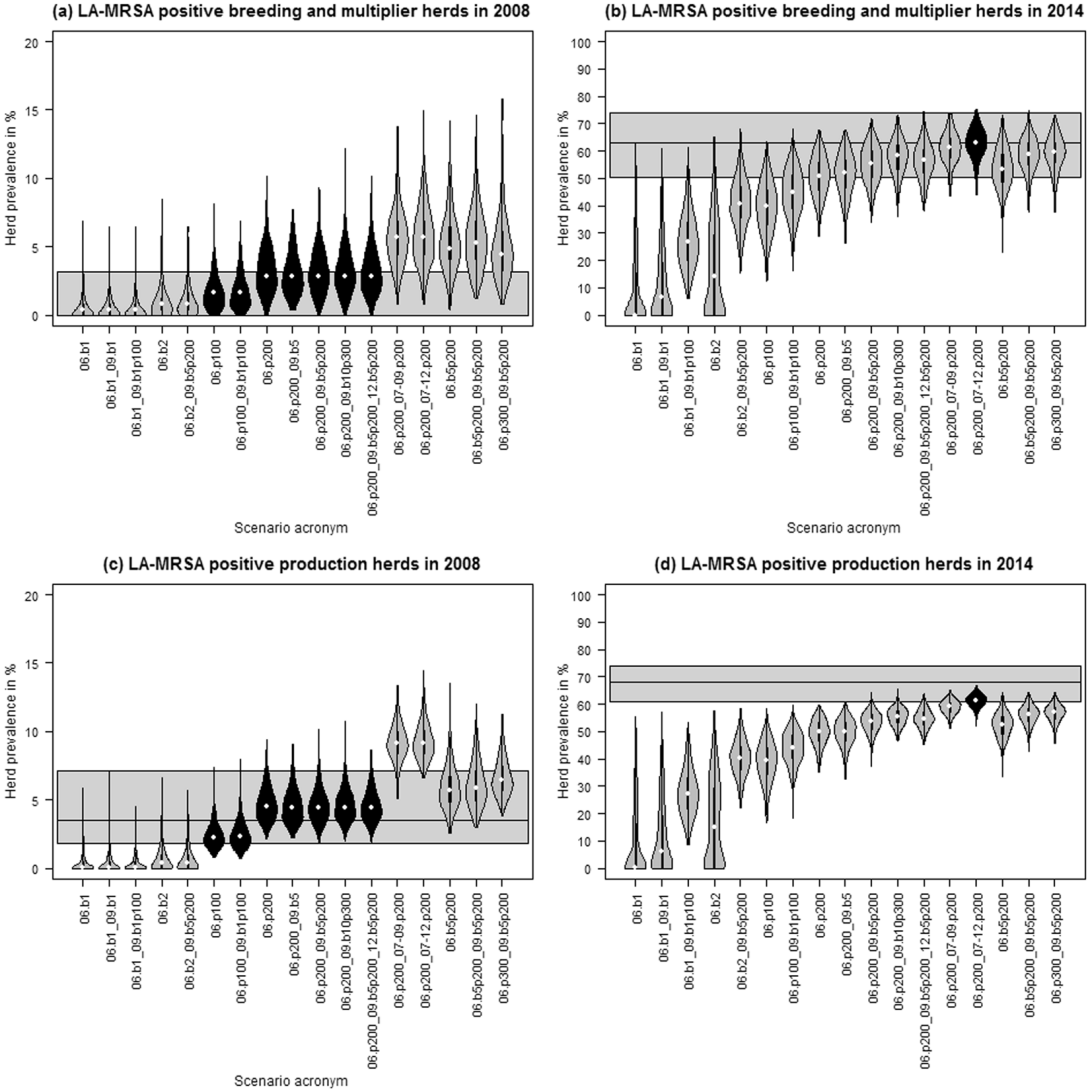
Herd prevalence in breeding and multiplier herds (**a**,**b**) and production herds (**c**,**d**) on 31^st^ December 2008 and 31^st^ December 2014 resulting from simulated LA-MRSA transmission via pig movements and indirect contacts, based on 500 iterations. Time-shifted transmission rates adapted from Broens *et al*.^28^ were used. The horizontal black line and grey area mark the observed prevalence and 95% confidence interval of the LA-MRSA screenings performed in Danish swine herds in 2008 and 2014. Black violins mark scenarios in which the simulated median prevalence in breeding and multiplier herds and production herds overlapped with the confidence interval of the LA-MRSA screening results. Example acronyms: 06.b5p200 – initialisation in five breeding and multiplier herds and 200 production herds in 2006, 06.b5_07-09.p200 – initialisation in five breeding and multiplier herds in 2006 and 200 production herds each year from 2007 to 2009.

In seven of the initialisation scenarios, the simulated median prevalence in breeding and multiplier herds as well as in production herds overlapped with the confidence interval of the LA-MRSA screening results for Danish pig herds in 2008 (Fig. 4). The median of five of the mentioned scenarios overlapped with the 2014 screening results for breeding and multiplier herds (Fig. 4b). However, for production herds, the median of only one scenario overlapped with the 2014 LA-MRSA screening results, whereas the median prevalence in 2008 exceeded the observed screening results (Fig. 4). The violin plots overlapped with the screening results for breeding and multiplier herds as well as for production herds in both 2008 and 2014 in eight initialisation scenarios, indicating that these scenarios might represent the spread of LA-MRSA among pig herds between 2006 and 2014.

### Sensitivity analysis

Based on the results, a default scenario of LA-MRSA initialisation in 100 production herds in 2006, and in one breeding and multiplier and 100 production herds in 2009 was used for the sensitivity analysis. The results of the scenarios with varied parameters were compared to the predicted median prevalence of the default scenario to allow the evaluation of the effects of parameter variation.

**Figure 5:**
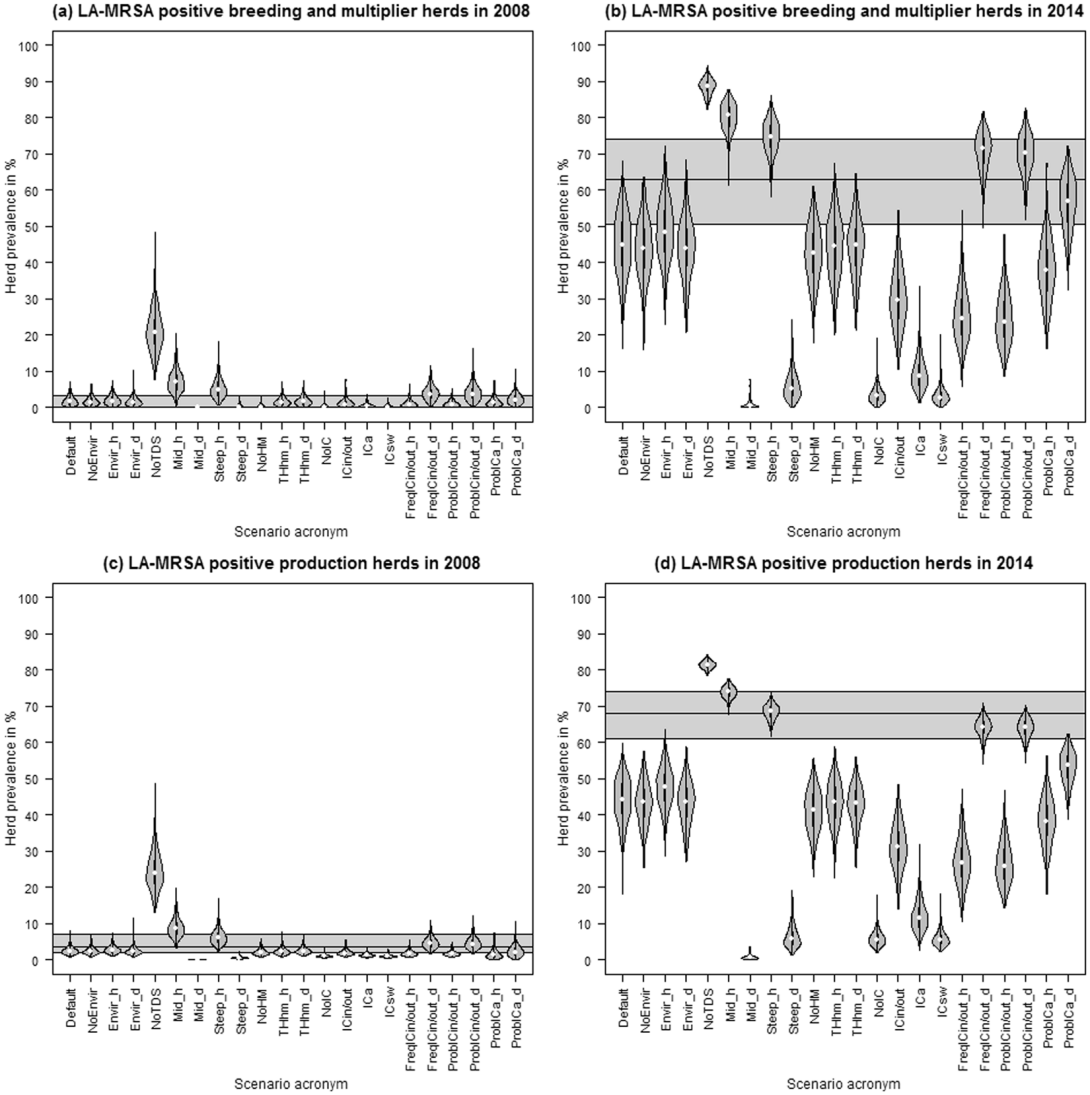
Sensitivity analysis showing the effect of changing the input for herd prevalence in breeding and multiplier herds (**a**,**b**) and production herds (**c**,**d**). Based on prevalence on 31^st^ December 2008 and 31^st^ December 2014 in 500 iterations. Time-shifted transmission rates adapted from Broens *et al*.^28^ were used. The horizontal black line and grey area mark the observed prevalence and 95% confidence interval of the LA-MRSA screenings performed in Danish swine herds in 2008 and 2014.

Scenarios relating to within-herd dynamics had a large impact on the model outcome. Running the model without time-dependent scaling of the transmission rates (NoTDS), and with halved midpoint (Mid_h) or doubled steepness (Steep_d) of the time-scaling function resulted in a higher prevalence compared to the standard scenario (Fig. 5). The modelled prevalence in the scenarios Mid_h and Steep_d fitted the LA-MRSA screening results from 2014 better than the default scenario. However, the modelled results for 2008 were above the LA-MRSA screening results. Compared to the default scenario, reducing the within-herd transmission by doubling the midpoint or halving the steepness of the time-scaling function decreased the modelled prevalence for breeding and multiplier herds and production herds for both years.

Scenarios representing environment-related recurrence (NoEnvir, Envir_h, Envir_d) and homogeneous mixing in small herds (NoHM, THhm_h, THhm_d) showed only small variations in the modelled outcome compared to the default scenario (Fig. 5).

Simulations that only modelled one of the implemented indirect transmission routes also resulted in a lower prevalence compared to the default scenario. Nevertheless, doubling (1) the average daily probability of indirect contact originating from an LA-MRSA-positive herd (variables *λ*_*in*_ and *λ*_*out*_, scenario FreqIC_in/out__d) or (2) the probability of infection via indirect contact from an LA-MRSA-positive herd (variables Prob_in_ and Prob_out_, scenario ProbIC_in/out__d) led to an increased simulated prevalence in 2008 and 2014 compared to the default scenario, as well as an overlap with the LA-MRSA screening results from 2014.

Violin plots for 2010 and 2011 are shown in Supplementary Information, Figure S8.

## Discussion

Livestock movements have been described as a driving factor in the spread of LA-MRSA, and the restriction of trade from MRSA-positive to MRSA-negative herds has therefore been discussed in the literature as a potential control option^32^. In order to assess whether a ban on animal movements would impede LA-MRSA spread, it is important to understand the extent to which it affects transmission. In the present study, the spread of LA-MRSA among pig herds via animal movements alone and via animal movements combined with indirect contact among pig herds was simulated to evaluate the effect of the modelled transmission routes. Several initialisation and sensitivity analysis scenarios were run and the results compared to the outcome of LA-MRSA screenings performed in Danish pig herds to externally validate the model and to evaluate the influence of the used model parameters on the model prediction. Based on assumptions in the model, pig movements alone cannot account for the spread of LA-MRSA in Denmark for the period 2006 to 2014. Even extensive initialisation in 2006 and subsequent years did not lead to the increase in LA-MRSA prevalence observed in Danish pig herds. Therefore, it is likely that a prevention strategy including only a ban on animal movements would not have prevented the spread of LA-MRSA among Danish pig herds.

Three routes were included to mimic the transmission of LA-MRSA via indirect contact: (1) human contacts, (2) abattoir movements, and (3) same owner. Modelling indirect contact clearly showed that the model was able to represent the observed prevalence in 2008, but showed lower predicted prevalence compared to that observed in 2014 for the default scenario (Fig. 4). Still, the figure clearly demonstrates the importance of indirect contact in the spread of LA-MRSA among pig herds compared to modelling the spread using only animal movements (Figs 3 and 4). The sensitivity analysis on the frequency and risk associated with indirect contact also showed its influential impact on the spread of LA-MRSA (Fig. 5). An LA-MRSA control program must therefore include biosecurity measures to limit the spread of LA-MRSA among herds via indirect contact. However, even though literature describing these transmission routes does exist^21,33,34^, detailed information on the frequency and effectiveness of LA-MRSA transmission via these indirect routes is lacking. As parameters related to indirect transmission routes were shown to be relevant for LA-MRSA spread among pig holdings, more knowledge should be gained to reduce uncertainties in the model outcome.

The model was initiated with many scenarios in an attempt to generate situations that would mimic the observed prevalence. The scenarios in which LA-MRSA was only initiated in herds in 2006 did not mimic the observed prevalence (Fig. 4). Nevertheless, randomly infecting herds in subsequent years resulted in an overlap between the some of the scenarios and the observed prevalence, indicating that the model is capable of replicating the LA-MRSA epidemic in Denmark between 2006 and 2014. As it is not possible to know exactly which (if any) of the modelled scenarios corresponded to reality, it can be difficult to choose the best scenario for future modelling of control measures. However, several scenarios can be modelled to identify the most effective strategy in different situations.

The fact that LA-MRSA had to be initialised in many herds over time suggests that it could not have spread solely via the simulated processes in this study (namely animal movements and indirect contacts), but that other processes must also have contributed. Humans can be persistent carriers of *S*. *aureus* and therefore be colonised for longer time periods^35^. In addition, humans working at or visiting a known LA-MRSA-positive herd might carry the pathogen for some time^36^ and might be able to transmit it to pigs in other visited herds. Therefore, the unexplained introduction of LA-MRSA to pig herds over time could represent introductions via humans. LA-MRSA could have been spread locally via for example, air, companion animals or rodents. However, to the best of our knowledge, there are no studies providing quantification of LA-MRSA transmission through these routes, and they were therefore not incorporated into the model framework.

Results of the sensitivity analysis showed that the parameters representing the within-herd spread of LA-MRSA had a substantial impact on model predictions (Fig. 5). This indicated that the within-herd prevalence could affect the between-herd prevalence and that reducing the prevalence within a herd might help to control LA-MRSA spread. This can be used as a starting point to study the impact of reducing the within-herd prevalence, for example by reducing antibiotic usage, and/or imposing higher biosecurity in a national control program.

Real data on pig herds and movements registered in the CHR were used as background data for the simulation^27^. Although the CHR provides very detailed data, it was necessary to estimate the age groups of moved pigs and, in some cases, the number of animals in a certain age group on the farm. Obligatory registration of the age of moved pigs in the CHR and automatic cross-validation between registers would increase the accuracy and validity of data in general as well as the accuracy of the results from this study.

It was necessary to exclude all trade herds due to missing information on the structure of the trade facilities. As there is no individual identification system for pigs, it was impossible to identify how long pigs might have been owned by traders or how they were mixed, thus potentially spreading LA-MRSA. However, there are very few trade herds in Denmark^13^ and this was therefore considered to be of limited importance.

Only one study simulating the spread of LA-MRSA among pig herds was found in the literature^37^. This study was conducted on pig herds in Denmark and it found that movement-induced transmission alone could yield a high probability of LA-MRSA persistence at a very low prevalence. However, the study used restricted movement data from only 1 year and did not include within-herd dynamics, which appeared to be influential in our study. In addition, indirect contact was modelled implicitly, without modelling the frequency or associated risk, and the impact of indirect contact on LA-MRSA spread was therefore not studied.

Besides individual-based Monte Carlo simulation models, also population-based models exist^25,38^. However, these population-based models simulate groups of individuals (herds), and actions such as pig movements or indirect contacts between herds would be applied to all herds in a certain group at the same time, which is not realistic. In contrast, individual-based models allow tracing of individual herds over time providing better insight into the modelled processes.

The model was developed to gain better insight into the spread of LA-MRSA among pig herds. However, it could be adapted to mimic the transmission of other pathogens that can be shed without clinical signs in pigs such as *Salmonella*. These pathogens could also be transmitted via animal movements and indirect contacts among pig herds. In addition, the model can also be used to simulate the spread of LA-MRSA in other farmed animal species. When applying the model to other pathogens or animal species, it may require adaptation according to the modelled disease or system. Modules mimicking other transmission pathways than the already implemented could be easily added to the original model.

In future, this model can be used to test control options to limit the spread of LA-MRSA. The effects of e.g. trade restrictions, as well as constraints related to indirect contact among herds could be evaluated to assist decision makers adapt local action plans. Additionally, the impact of increasing internal biosecurity and attempts to reduce antimicrobial usage at herd level could be evaluated. This could lead to the establishment of potential thresholds below which the within-herd prevalence would have to remain in order to have a clear impact on the between-herd spread of LA-MRSA.

### Code availability

The R script can be obtained from the authors upon request.

## Electronic supplementary material

Supplementary Information

## Data Availability

Data are owned by a third party. The authors had no special access privileges to these data. Danish pig movement data can be obtained for a fee by ordering an extract from https://chr.fvst.dk and by contacting the CGI Service Desk, tel. +45 70 21 13 21 or e-mailing dk-support.dk@cgi.com.
