# Supplementary material for: Drivers for Livestock-Associated Methicillin-Resistant Staphylococcus Aureus Spread Among Danish Pig Herds - A Simulation Study
Source: Sci Rep. 2018 Nov 16;8:16962. doi: 10.1038/s41598-018-34951-1 (PMC6240036; doi:10.1038/s41598-018-34951-1)
Supplement: Supplementary file 1 — Supplementary Information [file 41598_2018_34951_MOESM1_ESM.pdf]

# SUPPLEMENTARY INFORMATION

## DRIVERS FOR LIVESTOCK-ASSOCIATED METHICILLIN-RESISTANT STAPHYLOCOCCUS AUREUS SPREAD AMONG DANISH PIG HERDS - A SIMULATION STUDY

**Jana Schulz* -** Technical University of Denmark, National Veterinary Institute, Kemitorvet, Building 204, 2800 Kgs. Lyngby, Denmark, Tel: +45 35886167, E-Mail: janson@vet.dtu.dk

**Anette Boklund** – Technical University of Denmark, National Veterinary Institute, Kemitorvet, Building 204, 2800 Kgs. Lyngby, Denmark (anebo@vet.dtu.dk)

**Nils Toft** – Technical University of Denmark, National Veterinary Institute, Kemitorvet, Building 204, 2800 Kgs. Lyngby, Denmark (ntoft@vet.dtu.dk)

**Tariq Halasa** – Technical University of Denmark, National Veterinary Institute, Kemitorvet, Building 204, 2800 Kgs. Lyngby, Denmark (tahbh@vet.dtu.dk)

* Corresponding author

## Data preparation

*Herd information.* Holding information data included the holding and herd identification numbers. Several herds could be owned by the same farmer and therefore share an individual holding number, yet have different herd identification numbers. To keep the herds anonymous, new herd identification numbers were generated, and the herd was used as the unit of interest. Herds owned by the same farmer were marked to track the connection between them. The registered numbers of sows, weaners and finishers per year, as well as the herd type were extracted from the CHR.

The VetStat database records herd-level information on prescription-only drugs[1]. These data were used to add an antimicrobial usage index per year. The index was set to 1 if at least one prescription of tetracycline and/or beta-lactam was reported in the VetStat register.

In addition, the number of registered movements to abattoirs for each herd $h$ per year was calculated based on the movement data and this value divided by 365, giving a herd-specific $\lambda_a^h$ that was used as a parameter to model indirect contacts related to abattoir movements.

Our datasets lacked information on UTM coordinates for some of the herds. This information was updated using a website to calculate the coordinates based on the address of the holdings provided on the CHR website[2] (www.geoplaner.com/).

All herds were categorised according to: (1) herd type (breeding and multiplier herds, production herds, weaner herds, organic and free-range pig herds and hobby herds), (2) the proportion of sows, weaners and finishers registered in the CHR[2], and (3) the type of production (Table S1-S2).

No sows, weaners or finishers were registered in 181 herds in 2007 and 844 herds in 2014[3], but these herds had registered in-coming or out-going movements, and could therefore be considered active. The number of animals was estimated for these herds. For each herd type, the distribution of herds (with registered sows, weaners or finishers) was determined in each combination of categories 1-7 and A-C (Tables S1-S2). In addition, the average number of sows, weaners and finishers registered in these herds in the CHR was calculated for each category combination. To estimate the missing number of animals, a category combination was randomly assigned to herds that had not registered any animals. Based on this combination, the number of sows, weaners and/or finishers was calculated using an exponential distribution with lambda given by the average number of sows, weaners and finishers.

*Movement data.* Information on the movements of swine was available from the movement database, including the holding and herd identification numbers for both sending and receiving herds, the date of the pig movements and the number of pigs moved. No information was available on the age group (sows, weaners or finishers) of pigs moved.

The age group of pigs moved out of the sending herd and into the receiving herd was estimated based on the herd categories 1-7 and A-C (Tables S1-S4). If no weaners were registered, but were thought to have been moved out of a herd, they were assumed to have been moved directly from the sow section.

## Modelling disease spread within a herd

*Environment-related recurrence.* The probability decreases exponentially over time as follows:

$$f^{envir}(\Delta t) = \exp(-\alpha_{envir} \cdot \Delta t), \quad (1)$$

where $\Delta t$ is defined as the time difference between the current simulation day and the day when LA-MRSA died out in the respective herd.

*Within-herd dynamics.* For each individual susceptible animal, the probability of infection $Prob_{C_i}^t$ at each time step $t$ resulting from positive animals within compartment $C_i$ was assumed to be density-dependent and was calculated based on the number of LA-MRSA-positive pigs $I_{C_i}^t$ and the total number of pigs $N_{C_i}^t$ within the individual compartment as follows:

$$Prob_{C_i}^t = 1 - \exp\left(-\frac{\beta_{C_i}^t * I_{C_i}^t}{N_{C_i}^t}\right), \quad (2)$$

where $\beta_{C_i}^t$ is the daily transmission rate of the infection for each compartment $C_i$, and

$i \in \{sows, weaners, finishers\}$. The probability of infection $Prob_{C_iC_j}^t$ resulting from positive animals from another compartment $C_j \neq C_i$ was calculated as:

$$Prob_{C_iC_j}^t = 1 - \exp\left(-\frac{\beta_{C_iC_j}^t * I_{C_j}^t}{N_{C_j}^t}\right), \quad (3)$$

where $\beta^t_{C_iC_j}$ is the between-compartment transmission rate. The total probability for each individual in compartment $C_i$ was therefore:

$$Prob^t_{C_i} = 1 - (1 - Prob^t_{C_i}) * \prod_{j \neq i} (1 - Prob^t_{C_iC_j}). \quad (4)$$

In order to slow the within-herd spread, a time-dependent scaling function was introduced:

$$f(\Delta t) = 1 + exp\big(-k * (\Delta t - x)\big), \quad (5)$$

where $\Delta t$ is the simulation time since the introduction of LA-MRSA in the herd, and $k$ and $x$ are read-in parameters defining the steepness and the midpoint of the scaling function. The within-compartment transmission rates $\beta^t_{C_i}$ were divided by the scaling function. The time-shifted transmission rates led to a similar but delayed level of within-herd prevalence (Figures S1-S5).

Mimicking the within-herd dynamics in 100 large production herds for 365 days using the transmission rates adapted by Broens et al. (2012)[4] led to a median within-herd prevalence of 65% for herds using high-risk antibiotics and 59% for herds that did not use high-risk antibiotics (Figures S1-S4).

*Spread via indirect contacts.* The transmission between two herds $H_1$ and $H_2$ with the same owner was modelled using equation (2) to calculate the probability of infection $Prob^t_{H_iH_j}$ with $C_i = H_2$ and $C_j = H_1$. The number of LA-MRSA-positive pigs $N^t_{H_1}$ ($I^t_{H_1}$) was equivalent to the total number of LA-MRSA-positive sows, weaners and finishers in $H_1$. If $H_1$ was a large herd, the low-risk between-compartment transmission rate of $H_1$ was used, whereas if $H_1$ was small, the within-herd transmission rate of $H_1$ was used. If transmission was successful, new LA-MRSA-positive pigs were introduced to the sow compartment of herd $H_2$.

# FIGURES

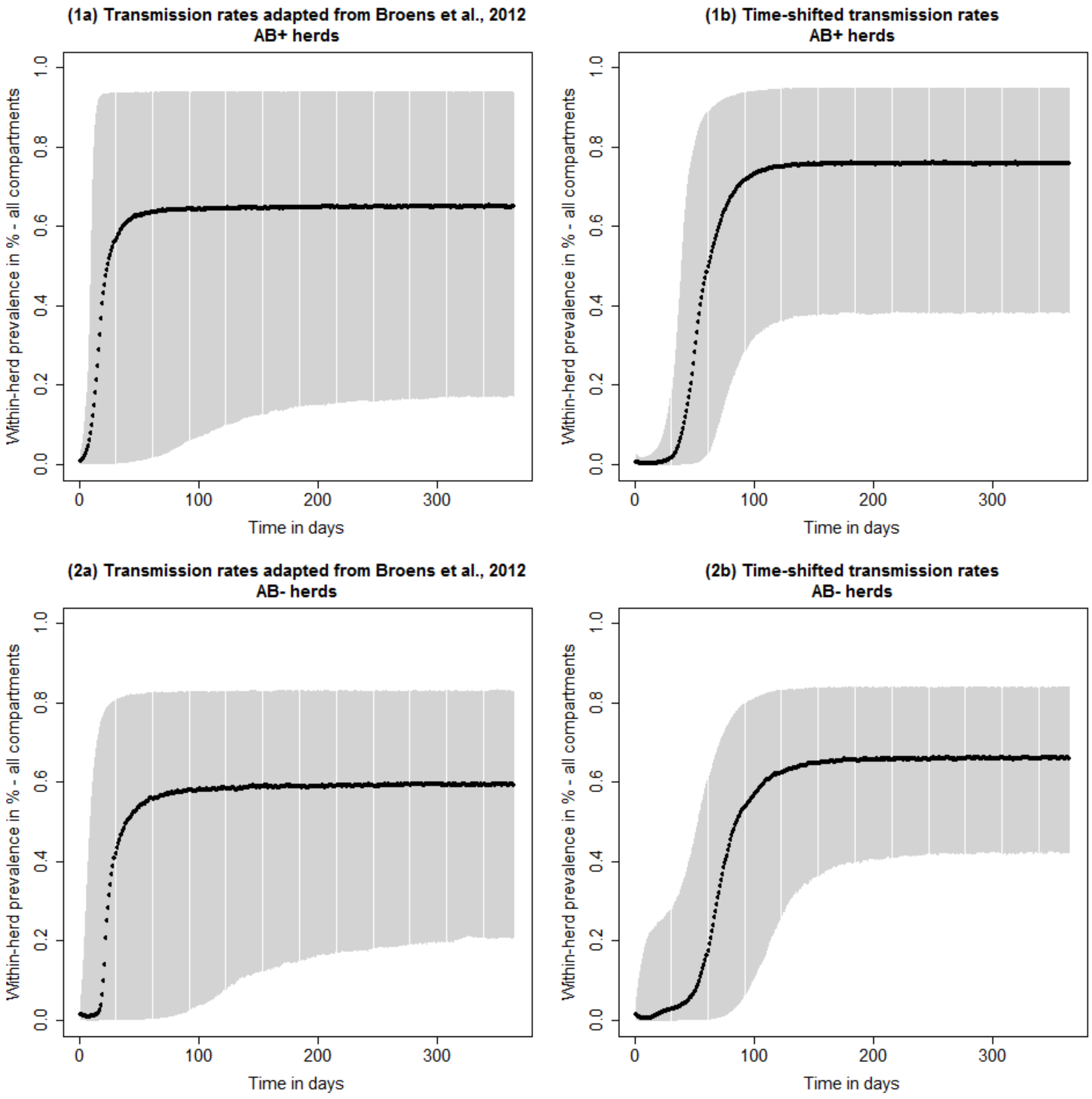


**Figure S1.** Development of the within-herd prevalence in large production herds in the first year after LA-MRSA initialisation in 100 production herds (1) using high-risk antibiotics, and (2) not using high-risk antibiotics for (a) transmission rates adapted from Broens et al.[4] (Table S5), and (b) time-shifted transmission rates. The black dotted line represents the median of 500 iterations and the grey area spans the 95% confidence interval.

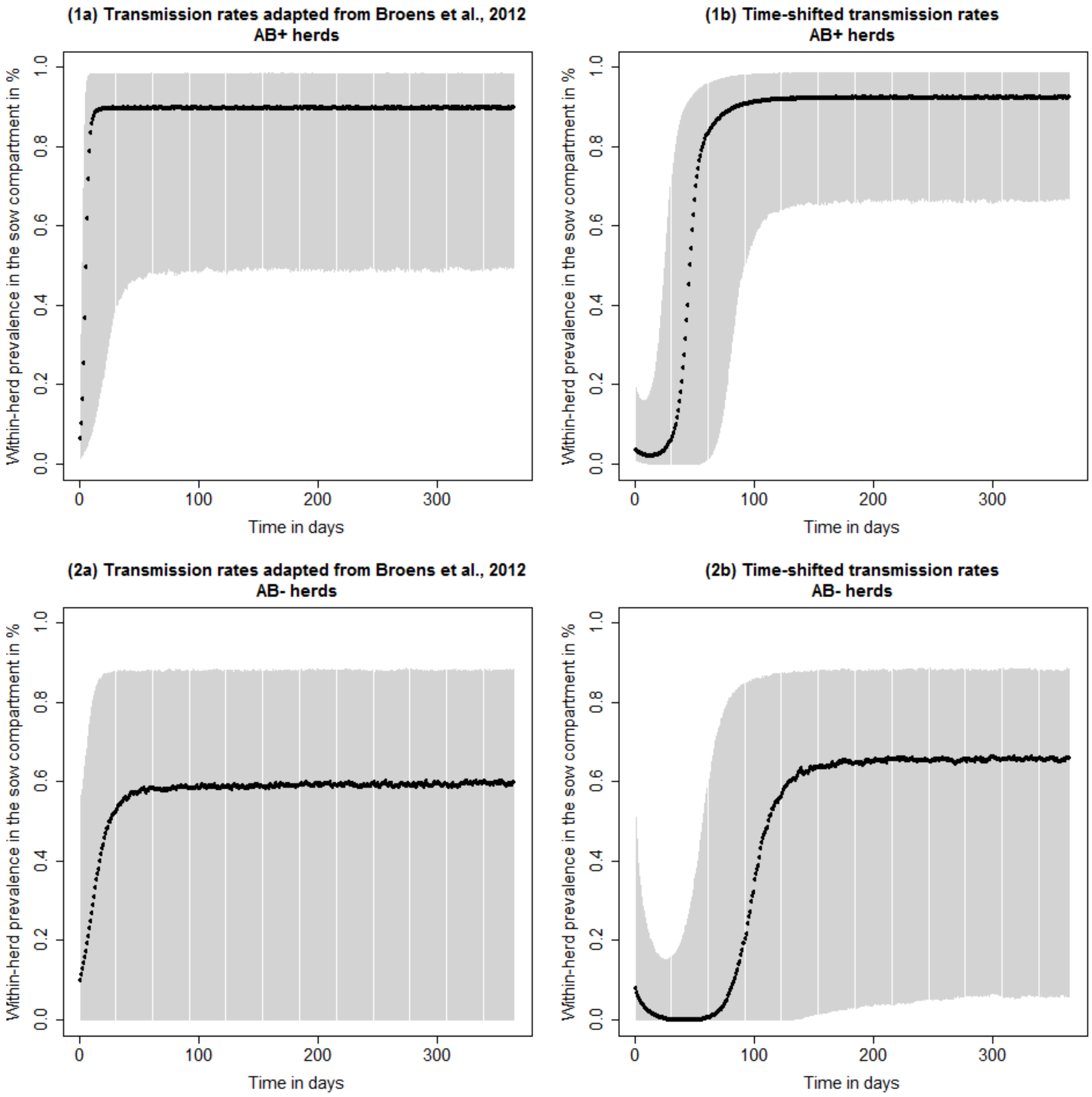


**Figure S2.** Development of the within-herd prevalence in the sow compartment in the first year after LA-MRSA initialisation in 100 production herds (1) using high-risk antibiotics, and (2) not using high-risk antibiotics for (a) transmission rates adapted from Broens et al.[4] (Table S5), and (b) time-shifted transmission rates. The black line represents the median of 500 iterations and the grey area spans the 95% confidence interval.

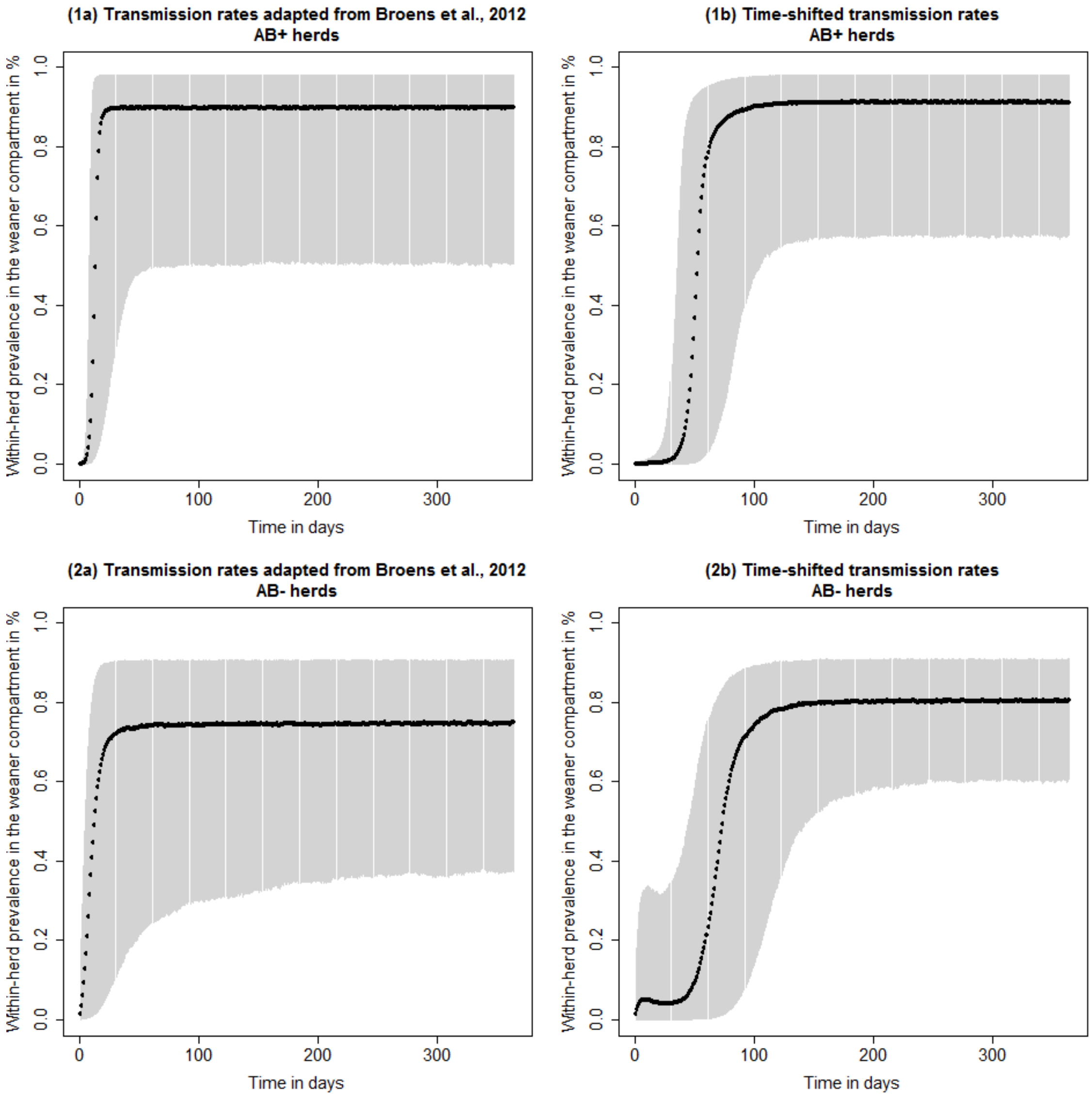


**Figure S3.** Development of the within-herd prevalence in the weaner compartment in the first year after LA-MRSA initialisation in 100 production herds (1) using high-risk antibiotics, and (2) not using high-risk antibiotics for (a) transmission rates adapted from Broens et al.[4] (Table S5), (b) time-shifted transmission rates, and (3) time-shifted and scaled transmission rates. The black line represents the median of 500 iterations and the grey area spans the 95% confidence interval.

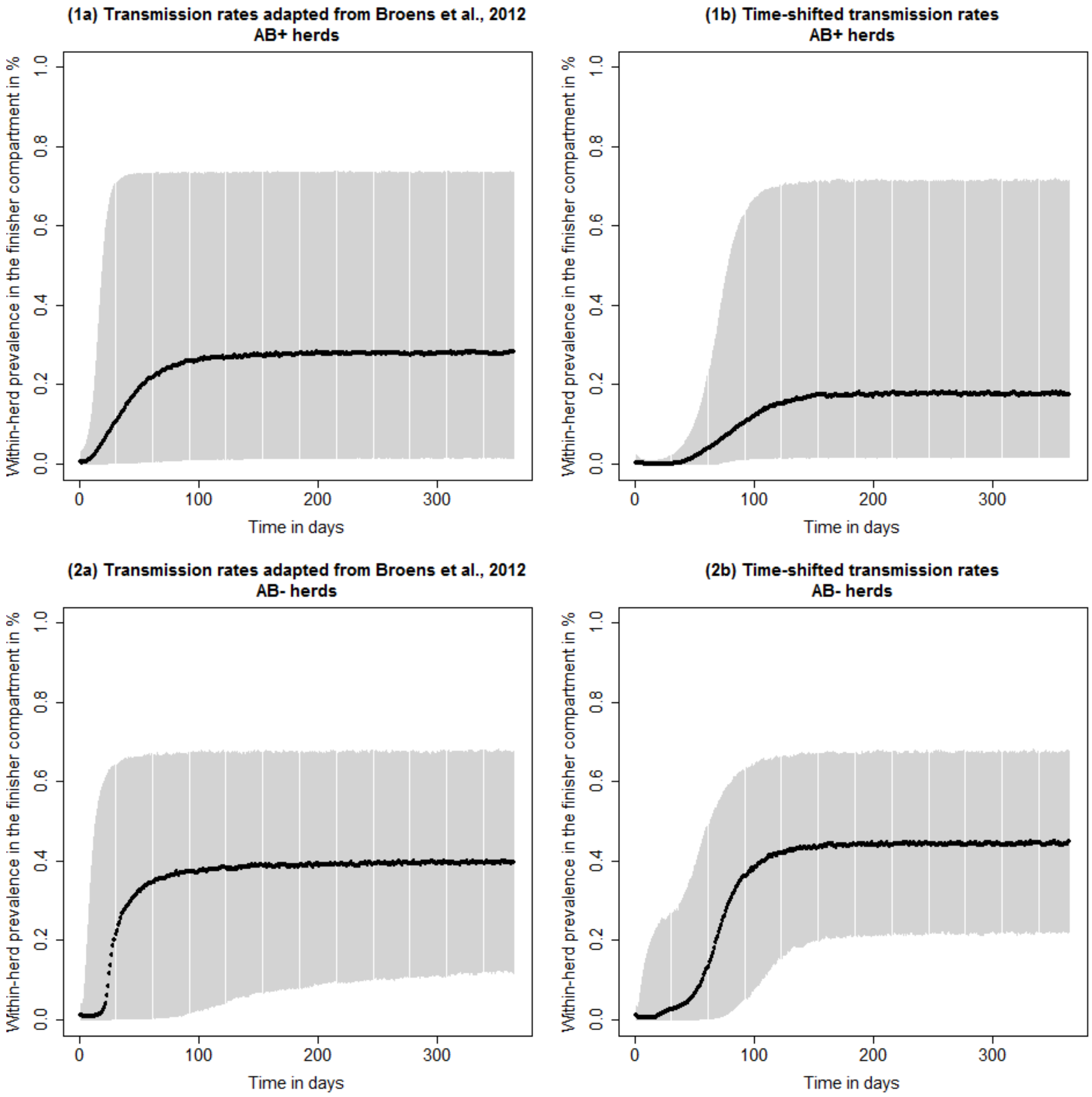


**Figure S4.** Development of the within-herd prevalence in the finisher compartment in the first year after LA-MRSA initialisation in 100 production herds (1) using high-risk antibiotics, and (2) not using high-risk antibiotics for (a) transmission rates adapted from Broens et al.[4] (Table S5), and (b) time-shifted transmission rates. The black line represents the median of 500 iterations and the grey area spans the 95% confidence interval.

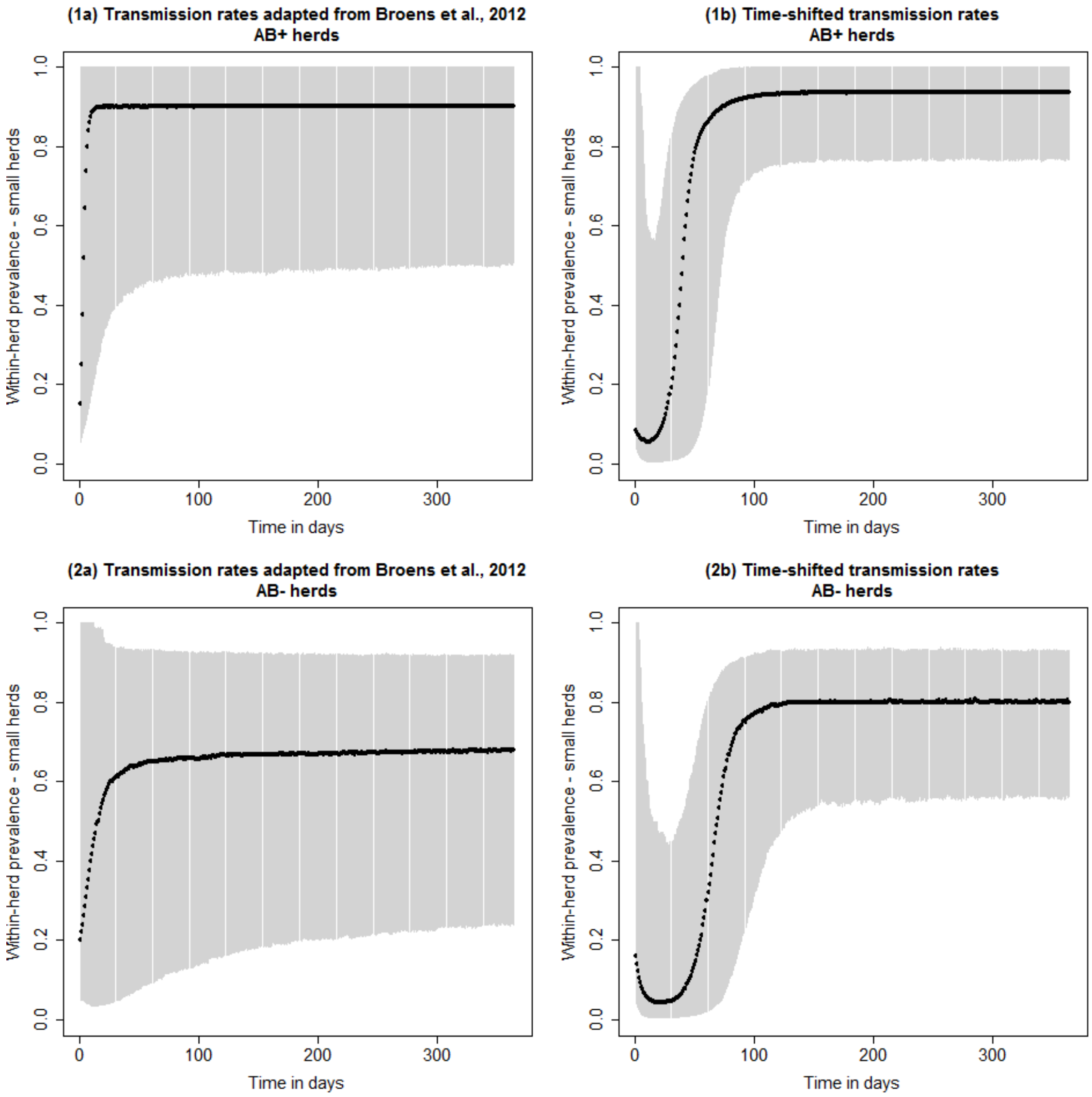


**Figure S5.** Development of the within-herd prevalence in small herds in the first year after LA-MRSA initialisation in 100 production herds (1) using high-risk antibiotics, and (2) not using high-risk antibiotics for (a) transmission rates adapted from Broens et al.[4] (Table S5), and (b) time-shifted transmission rates. The black line represents the median of 500 iterations and the grey area spans the 95% confidence interval.

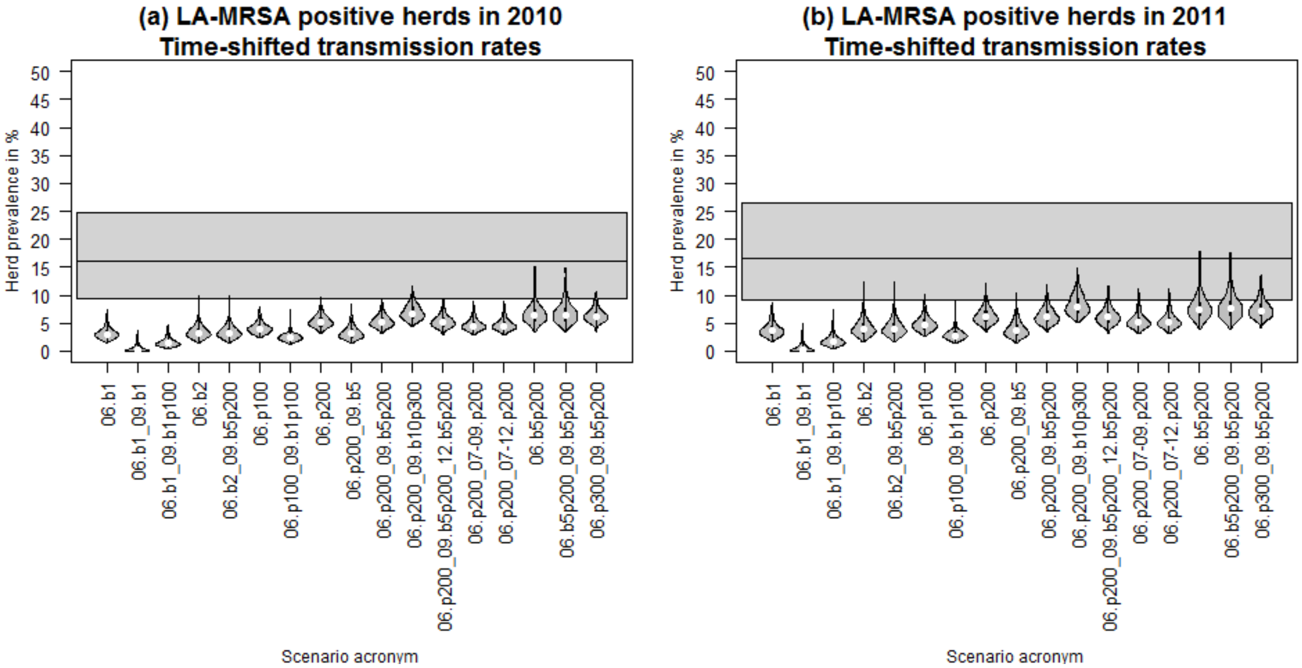


**Figure S6.** Violin plots summarising the herd prevalence in all herds in 2010 and 2011 based on 500 iterations of 17 LA-MRSA initialisation scenarios using time-shifted transmission rates adapted from Broens et al.[4], and modelling transmission only via animal movements.

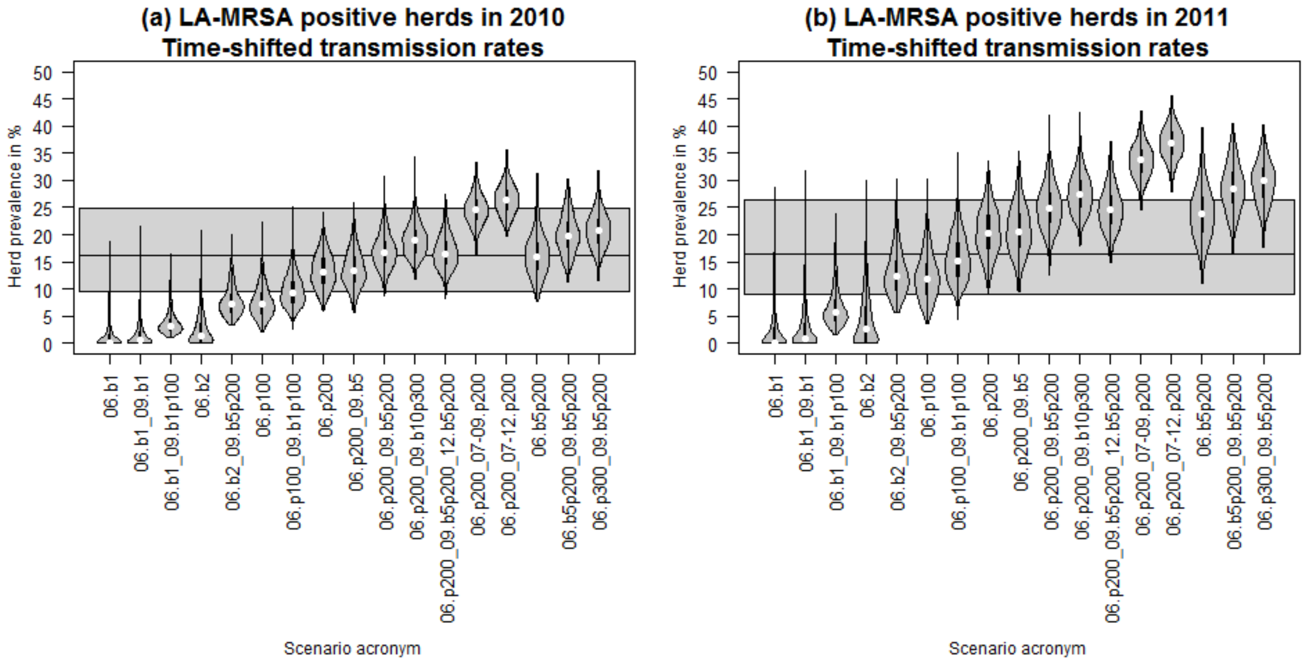


**Figure S7.** Violin plots summarising the herd prevalence in all herds in 2010 and 2011 based on 500 iterations of 17 LA-MRSA initialisation scenarios using time-shifted transmission rates adapted from Broens et al.[4], and modelling transmission via animal movements and via indirect contacts between herds.

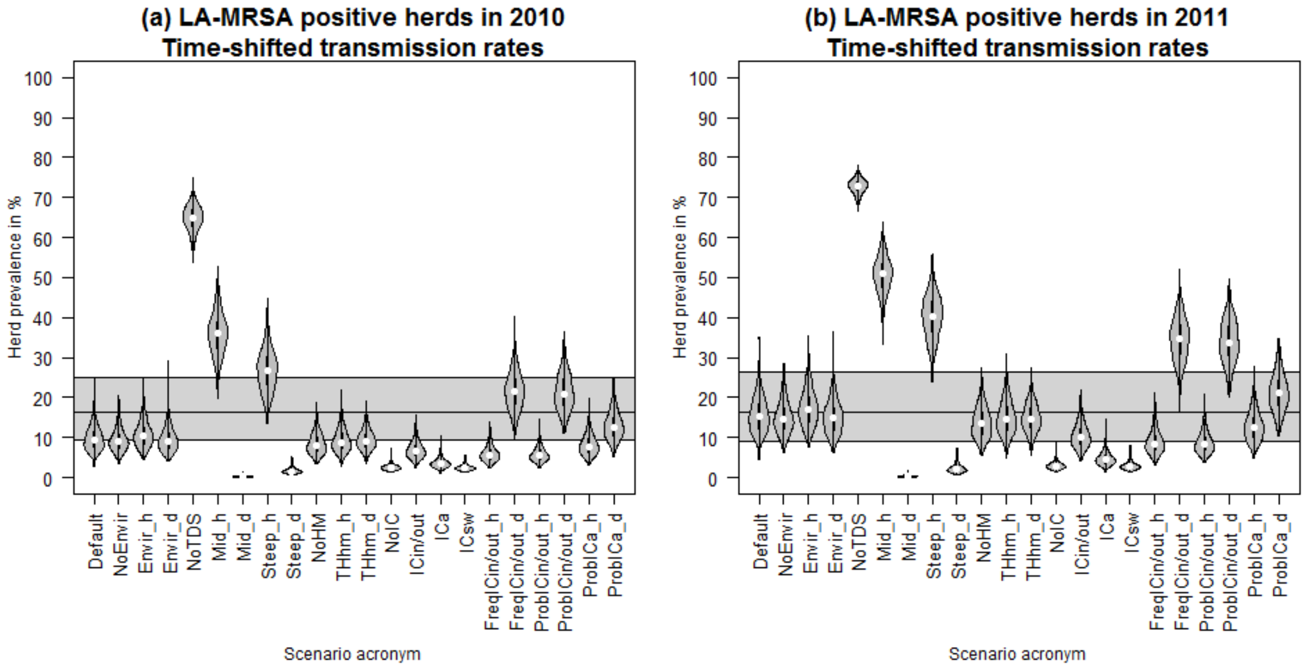


**Figure S8.** Violin plots summarising the herd prevalence in all herds in 2010 and 2011 based on 500 iterations of 21 LA-MRSA sensitivity analysis scenarios using time-shifted transmission rates adapted from Broens et al.[4], and modelling transmission via animal movements and via indirect contact among herds. The first violin represents the results of the default initialisation scenario 06.b1_09.b1p100.

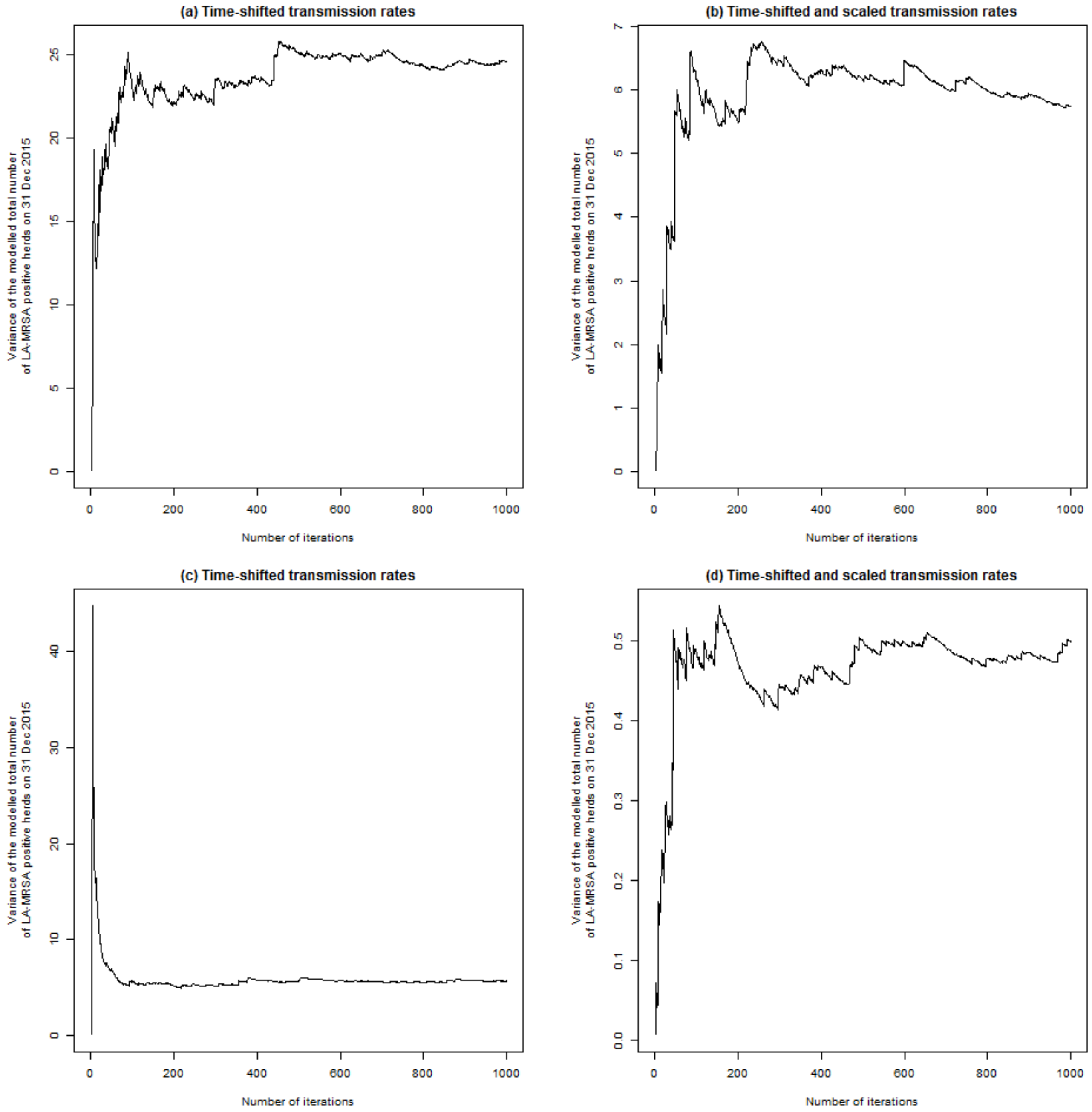


**Figure S9.** Variance of the total number of LA-MRSA-positive herds on 31[st] December 2015 against the number of simulation repetitions. Convergence is assumed when the variance stabilises. All convergence plots refer to the scenario with LA-MRSA initialisation in 100 production herds in 2006 and re-introduction for one breeding and multiplier herd and 100 production herds in 2009. (a) and (b) represent scenarios including indirect transmission routes, while (c) and (d) represent the results for transmission via pig movements only.

# TABLES

**Table S1.** Herd categories related to the combination of sows, weaners, and finishers registered in the CHR, used to estimate the number of sows, weaners and finishers in herds that did not register any pigs.

| Category | Sows registered | Weaners registered | Finishers registered |
|---|---|---|---|
| **1** | yes | yes | yes |
| **2** | yes | yes | no |
| **3** | yes | no | yes |
| **4** | no | yes | yes |
| **5** | yes | no | no |
| **6** | no | yes | no |
| **7** | no | no | yes |

**Table S2.** Herd categories related to the type of production based on the registered number of sows and finishers.

| Category | Type of production | Definition |
|---|---|---|
| **A** | Finisher herd | > 7.5 finishers per sow |
| **B** | Sow herd | < 5 finishers per sow |
| **C** | Integrated herd | 5 – 7.5 finishers per sow |

**Table S3.** Assumed age group (s = sows, w = weaners, f = finishers) of pigs moved **out** of the sending herd / **in** to the receiving herd. The letter x is used when the movement type is dependent on a defined threshold (Table S4). Below or equal to this threshold, finishers are assumed to be moved out of the sending herd and sows are assumed to be moved in to the receiving herd. Above this threshold, it is assumed that weaners/finishers were moved in to / out of the sending / receiving herd.

| | Herd category of receiving herd | | | | | | | | | | |
|---|---|---|---|---|---|---|---|---|---|---|---|
| Herd category of sending herd | 1A | 1B | 1C | 2A | 2B | 2C | 3B | 4A | 5B | 6A | 7C |
| 1A | w/f | f/s | f/s | w/f | f/s | f/s | f/s | w/f | f/s | w/f | w/w |
| 1B | f/s | f/s | f/s | f/s | f/s | f/s | f/s | w/f | f/s | w/f | w/w |
| 1C | x | x | x | x | x | x | f/s | w/f | f/s | w/f | w/w |
| 2A | w/f | f/s | f/s | w/f | f/s | f/s | f/s | w/f | f/s | w/f | w/w |
| 2B | f/s | f/s | f/s | f/s | f/s | f/s | f/s | w/f | f/s | w/f | w/w |
| 2C | x | x | x | x | x | x | f/s | w/f | f/s | w/f | w/w |
| 3B | w/f | w/f | w/f | w/f | w/f | w/f | w/s | w/f | w/s | w/f | w/w |
| 4A | w/f | w/f | w/f | w/f | w/f | w/f | w/s | w/f | w/s | w/f | w/w |
| 5B | w/f | w/f | w/f | w/f | w/f | w/f | w/s | w/f | w/s | w/f | w/w |
| 6A | f/f | f/f | f/f | f/f | f/f | f/f | f/s | f/f | f/s | f/f | f/w |
| 7C | w/f | w/f | w/f | w/f | w/f | w/f | w/s | w/f | w/s | w/f | w/w |

**Table S4.** Assumed thresholds for setting the age group of movements if the movement type is given as *x* in Table S3. The thresholds are dependent on the registered herd type and for production, organic and hobby herds on the size of the herd. In small herds, homogeneous mixing of pigs is assumed, whereas separate compartments for the three age groups (sows, weaners, and finishers) were modelled for large herds. The number of pigs assumed to constitute a small herd was set to 200 animals.

| Threshold for movement types | Production (small) | Organic (small) | Breeding and multiplier | Weaner | Hobby (small) | Production (large) | Organic (large) | Hobby (large) |
|---|---|---|---|---|---|---|---|---|
| **Breeding sites** | | | | | | | | |
| Breeding and multiplier herds | 3 | 3 | 50 | 20 | 3 | 50 | 50 | 50 |
| **Production sites** | | | | | | | | |
| Production (small) | 3 | 3 | 3 | 3 | 3 | 3 | 3 | 3 |
| Production (large) | 3 | 3 | 20 | 20 | 3 | 20 | 20 | 20 |
| Organic (small) | 3 | 3 | 3 | 3 | 3 | 3 | 3 | 3 |
| Organic (large) | 3 | 3 | 20 | 20 | 3 | 20 | 20 | 20 |
| Weaner herds | 3 | 3 | 3 | 3 | 3 | 3 | 3 | 3 |
| **Hobby sites** | | | | | | | | |
| Hobby herds (small) | 3 | 3 | 3 | 3 | 3 | 3 | 3 | 3 |
| Hobby herds (large) | 3 | 3 | 20 | 20 | 3 | 20 | 20 | 20 |

**Table S5.** Assumed values for a PERT distribution to define herd-specific cure rates and transmission rates based on the use of high-risk antibiotics, adapted by Broens et al.[4] The most likely (mode) and assumed minimum and maximum values for the PERT distributions were calculated based on values for $R_0$ (and their 95% CI) resulting from multivariable analysis of Dutch data, with 10.3 days taken to be the duration of the infectious period[5]. PERT distributions were defined as transformation of the Beta distribution with minimum (min), maximum (max) and most likely value (mode) and a mean $\mu = \frac{\text{min}+4\cdot\text{mode}+\text{max}}{6}$.

| Use of high-risk antibiotics | Cure rate (all herds) | Within-compartment transmission rate | Low-risk between-compartment transmission rate | High-risk between-compartment transmission rate | Transmission rate in small herds |
|---|---|---|---|---|---|
| no | min = 0.056, max = 0.385, mode = 0.097 | min = 0.111<br>max = 0.856<br>mode = 0.307 | min = 0.00175<br>max = 0.00301<br>mode = 0.00233 | min = 0.07184<br>max = 0.48155<br>mode = 0.18301 | min = 0.111<br>max = 0.856<br>mode = 0.307 |
| yes | | min = 0.211<br>max = 2.924<br>mode = 0.784 | min = 0.00330<br>max = 0.01029<br>mode = 0.00583 | min = 0.13689<br>max = 1.64515<br>mode = 0.46796 | min = 0.211<br>max = 2.924<br>mode = 0.784 |

**Table S6.** Distance probabilities of indirect contact among herds for the different types of contact and distance categories.

| Distance category (up to … km) | Contact from indoor herd | Contact from outdoor herd | Contact related to abattoir movements |
|---:|---:|---:|---:|
| 1 | 0.12 | 0.12 | 0.0054 |
| 3 | 0.24 | 0.24 | 0.0224 |
| 10 | 0.30 | 0.29 | 0.1898 |
| 15 | 0.083 | 0.089 | 0.1503 |
| 20 | 0.083 | 0.089 | 0.1252 |
| 30 | 0.054 | 0.050 | 0.1747 |
| 40 | 0.01714 | 0.01714 | 0.1347 |
| 50 | 0.01714 | 0.01714 | 0.0823 |
| 60 | 0.01714 | 0.01714 | 0.0454 |
| 70 | 0.01714 | 0.01714 | 0.0274 |
| 80 | 0.01714 | 0.01714 | 0.0141 |
| 90 | 0.01714 | 0.01714 | 0.0099 |
| 100 | 0.01714 | 0.01714 | 0.0108 |
| 110 | 0 | 0 | 0.0041 |
| 120 | 0 | 0 | 0.0025 |
| 130 | 0 | 0 | 0.00018 |
| 140 | 0 | 0 | 0.00006 |
| 150 | 0 | 0 | 0.00006 |
| 160 | 0 | 0 | 0.00006 |
| 170 | 0 | 0 | 0.00006 |
| 180 | 0 | 0 | 0.00006 |
| 190 | 0 | 0 | 0.00006 |
| 200 | 0 | 0 | 0.00006 |
| 210 | 0 | 0 | 0.00006 |
| 220 | 0 | 0 | 0.00006 |
| 230 | 0 | 0 | 0.00006 |
| 240 | 0 | 0 | 0.00006 |
| 250 | 0 | 0 | 0.00006 |
| 350 | 0 | 0 | 0.00006 |
| 1,000 | 0 | 0 | 0.00006 |

**Table S7.** LA-MRSA initialisation scenarios tested using the developed simulation model.

| Acronym | First introduction of LA-MRSA in the simulation model | Initialisation of LA-MRSA-positive herds at later points in time |
|---|---|---|
| 06.b1 | **2006:**<br>- 1 breeding and multiplier herd | none |
| 06.b1_09.b1 | | **2009:**<br>- 1 breeding and multiplier herd |
| 06.b1_09.b1p100 | | **2009:**<br>- 1 breeding and multiplier herd<br>- 100 production herds |
| 06.b2 | **2006:**<br>- 2 breeding and multiplier herds | none |
| 06.b2_09.b5p200 | | **2009:**<br>- 5 breeding and multiplier herds<br>- 200 production herds |
| 06.p100 | **2006:**<br>- 1% of production herds (100 herds) | none |
| 06.p100_09.b1p100 | | **2009:**<br>- 1 breeding and multiplier herd<br>- 100 production herds |
| 06.p200 | **2006:**<br>- 2% of production herds (200 herds) | none |
| 06.p200_09.b5 | | **2009:**<br>- 5 breeding and multiplier herds |
| 06.p200_09.b5p200 | | **2009:**<br>- 5 breeding and multiplier herds<br>- 200 production herds |
| 06.p200_09.b10p300 | | **2009:**<br>- 10 breeding and multiplier herds<br>- 300 of production herds |
| 06.p200_09.b5p200_ 12.b5p200 | | **2009:**<br>- 5 breeding and multiplier herds<br>- 200 production herds<br>**2012:**<br>- 5 breeding and multiplier herds<br>- 200 production herds |
| 06.p200_07-09.p200 | | **2007-2009:**<br>- 200 production herds each year |
| 06.p200_07-12.p200 | | **2007-2012:**<br>- 200 production herds each year |
| 06.b5p200 | **2006:**<br>- 2% of production herds (200 herds)<br>- 2% of breeding and multiplier herds (5 herds) | none |
| 06.b5p200/09.b5p200 | | **2009:**<br>- 5 breeding and multiplier herds<br>- 200 production herds |
| 06.p300/09.b5p200 | **2006:**<br>- 3% of production herds (300 herds) | **2009:**<br>- 5 breeding and multiplier herds<br>- 200 production herds |

## REFERENCES


1. Stege, H., Bager, F., Jacobsen, E. & Thougaard, A. VETSTAT-the Danish system for surveillance of the veterinary use of drugs for production animals. *Prev. Vet. Med.* **57,** 105–15 (2003).

2. Danish Veterinary and Food Administration. Central Husbandry Register (CHR). (2015). at <https://www.foedevarestyrelsen.dk/english/Animal/AnimalHealth/Central_Husbandry_Register/Pages/default.aspx>

3. Schulz, J., Boklund, A., Halasa, T. H. B., Toft, N. & Lentz, H. H. K. Network analysis of pig movements : Loyalty patterns and contact chains of different holding types in Denmark. *PLoS One* **12,** e0179915 (2017).

4. Broens, E. M. *et al.* Longitudinal study on transmission of MRSA CC398 within pig herds. *BMC Vet. Res.* **8,** 58 (2012).

5. Broens, E. M., Graat, E. A. M., van de Giessen, A. W., Broekhuizen-Stins, M. J. & de Jong, M. C. M. Quantification of transmission of livestock-associated methicillin resistant Staphylococcus aureus in pigs. *Vet. Microbiol.* **155,** 381–388 (2012).
